# Early detection of central lung cancer: are we there yet?

**DOI:** 10.1186/s12931-015-0212-8

**Published:** 2015-04-23

**Authors:** Ali I Musani, Akrum Al-Zubaidi

**Affiliations:** Professor of Medicine National Jewish Health and University of Colorado, Denver, Colorado USA; Interventional Pulmonary Fellow, National Jewish Health, Denver, Colorado and Valley View Hospital, Glenwood Springs, CO USA

Computed Tomography (CT), Magnetic Resonance Imaging (MRI), and X-rays have been very disappointing in the detection and diagnosis of central (airways/endobronchial) lesions.

Bronchoscopy with direct visualization and other supportive technologies has been the primary tool for the detection and surveillance of endobronchial precancerous and cancerous lesions. Early detection of lung cancer allows early interventions which has a survival benefit as shown by a 20% relative decrease in lung cancer specific mortality with low-dose computed tomography screening and high-risk groups [1]. CT screening of the thorax is able to detect lesions that are peripheral, central, and sub centimeter, however it is insensitive for detection of precancerous and early small cancerous lesions arising from the airways.

The pathological progression from normal bronchogenic epithelium to squamous metaplasia followed by dysplasia and finally carcinoma in situ(CIS) is supported by sputum cytology studies as well as animal data [2,3], however other models such as multiple foci of precursor lesions that are produced throughout the respiratory epithelium may develop into carcinoma in situ as opposed to a stepwise progression of a single area [4]. Regardless, prompt detection of high-risk patients that offers early diagnosis of preinvasive lesions could allow for early intervention and improved survival.

The use of white light bronchoscopy is the standard imaging tool for the diagnosis of central airway tumors. However, in the detection of precancerous lesions WLB has a low sensitivity and specificity [5,6]. Recent advances have allowed bronchoscopists to evaluate the airway with advanced high-resolution imaging modalities. The development of the ideal bronchoscopic imaging technique capable of detecting preinvasive lesions is ongoing.

Currently autofluorescence bronchoscopy (AFB), narrow-band imaging(NBI), high magnification bronchovideoscopy (HMB), probe based confocal endomicroscopy (pCLE), optical coherence tomography (OCT), radial probe endobronchial ultrasonography (R-EBUS) and now high definition bronchoscopy (HD) with surface enhancement (i-scan) are all being studied or used in clinical practice to evaluate the airway. The ideal system would be able to detect and define preinvasive lesions so that not only are we able to preemptively treat these lesions but determine effectiveness of treatment with noninvasive or minimally invasive techniques.

In this issue of Respiratory Research, van der Heijden and colleagues report a randomized blinded prospective study, which examined the use of 5 videobronchoscopy techniques in 29 patients in which the authors explore whether different modalities might lead to improved detection of suspicious vascular changes or suspected preinvasive lesions.

In the previous literature, WLB combined with AFB increased the diagnostic accuracy for squamous dysplasia, CIS, and early lung cancer with a high sensitivity but resulted in a large number of false positives and unneeded biopsies [7,8]. Heijden and colleagues did show that HD bronchoscopy with i-scan improve detection of vascular changes over WLB and AFB however AFB did detect more preinvasive lesions over WLB and HD modes. Histological confirmation was not obtained in this study so it is difficult to discern whether there would be improved accuracy of HD modes over AFB. That said we agree that it is likely that improved images and image enhancement which are able to better identify vascular abnormalities could improve overall diagnostic yield.

Further studies in which a potential combination of methods, which improve efficiency of the airway evaluation while also improving accuracy of diagnosis are needed to fully determine the clinical implications of these modalities. Finally, the bigger question remains, would early detection and treatment of precancerous lesions in the airway as a primary or a recurrent lesion changes the ultimate outcomes in patients.
